# Protective effect of selenomethionine on T-2 toxin-induced liver injury in New Zealand rabbits

**DOI:** 10.1186/s12917-021-02866-1

**Published:** 2021-04-09

**Authors:** Yumei Liu, Haojie Wang, Mengyu Zhang, Jiajia Wang, Zhixiang Zhang, Yuqin Wang, Yingying Sun, Ziqiang Zhang

**Affiliations:** 1 College of Animal Science and Technology, Henan University of Science and Technology, Luoyang, 471000 Henan China; 2 College of Life Science, Yangtze University, Jingzhou, 434023 Hubei China; 3 Engineering Research Center for Mutton Sheep Breeding of Henan Province, Luoyang, 471000 Henan China

**Keywords:** Apoptotic, Liver, Oxidative stress, Selenomethionine, T-2 toxin

## Abstract

**Background:**

T-2 toxin is a mycotoxin produced by Fusarium species that is highly toxic to animals. Recent studies have indicated that Selenomethionine (SeMet) have protective effect against mycotoxins-induced toxicity. The aim of the present study was to investigate the protective effect of SeMet on T-2-toxin-induced liver injury in rabbit and explore its molecular mechanism. Fifty rabbits (30 d, 0.5 ± 0.1 kg) were randomly divided into 5 groups: control group, T-2 toxin group, low, medium and high dose SeMet treatment group. The SeMet-treated group was orally pretreated with SeMet (containing selenium 0.2 mg/kg, 0.4 mg/kg and 0.6 mg/kg) for 21 days. On the 17th day, T-2 toxin group and SeMet-treated group were orally administered with T-2 toxin (0.4 mg/kg body weight) for 5 consecutive days.

**Results:**

The results showed that low-dose SeMet significantly improved T-2 toxin-induced liver injury. We found that low-dose SeMet can reduce the level of oxidative stress and the number of hepatocyte apoptosis. Moreover, the levels of Bax, caspase-3 and caspase-9 were significantly reduced and the levels of Bcl-2 were increased.

**Conclusions:**

Therefore, we confirmed that low-dose SeMet may protect rabbit hepatocytes from T-2 toxin by inhibiting the mitochondrial-caspase apoptosis pathway.

## Background

T-2 toxin is a secondary metabolite produced by different species of Fusarium including F. soprotrichioides, *F. poae* and F. acuinatum, which can infect corn, wheat, barley, rice and other crops in the field and in storage under tropical climate or humid storage conditions [1]. It has extremely high chemical stability under changing environmental conditions, so that autoclaving is not easy to inactivate T-2 toxins during feed production and processing. When livestock consume animal feed containing the T-2 toxin, it can cause a variety of toxicity, such as hemotoxicity, immunotoxicity and genotoxicity [2]. Food and feed-stuffs prepared using mycotoxin-containing crops deterio-rate nutritional content and represent a potential risk for animal and human health [3].

As the main place of substance metabolism, the liver is the main target of the toxicity and the metabolite of the T-2 toxin [4]. Exposure to T-2 causes a significant reduction in serum total protein (TP) and marked increases in the activities of transaminases, such as aspartate aminotransferase (AST) and alanine aminotransferase (ALT) [4]. Accumulated studies have demonstrated that oxidative stress is the main mechanism of toxicity of T-2 toxin [5]. Oxidative stress [6] is characterized by down-regulation of total antioxidant capacity (T-AOC) and antioxidant enzyme activities, including catalase (CAT), glutathione peroxidase (GSH-Px), and superoxide dismutase (SOD), these are all part of the antioxidant reserve. The decreased antioxidant capacity leads to the overproduction of reactive oxygen species (ROS), which damages the cellular components and induces cell apoptosis. In addition, after entering the liver, T-2 toxin can also inhibit the expression and activity of certain liver drug metabolism enzymes by inhibiting hepatocyte protein synthesis [7]. For example, after exposure to T-2 toxin for 13 days, the activities of liver microsomal cytochrome P450 decreased [8, 9]. Similarly, the expression of drug metabolizing enzyme P4501A gene was decreased after exposure to T-2 toxin in pigs [10].

T-2 toxins are widely distributed in nature. Contamination of animal feed with mycotoxins still occurs very often [3], In a ten-year survey of the global mycotoxin content of feed conducted by Biomin, 19% of the samples were found to be contaminated with T-2 toxin [11]. The contamination rate of T-2 toxin in feedstuff samples in China was 79.5%, and these samples had the highest levels of T-2 toxin, at up to 735 ng/g [12]. Rabbit feed is vulnerable to contamination by T-2 toxins due to widespread contamination of grains by T-2 toxins and improper storage of feed. Rabbits can be considered to be rather sensitive to T-2 toxin, as reflected by the relatively low (1.1 mg/kg BW) LD50 values [13], presumably due to the re-consumption of the toxin-containing caecal content by caecotrophy [14]. And there are literatures showing that rabbits are susceptible to a sub-acute T-2 mycotoxicosis [15, 16], causing great economic losses to the animal husbandry industry. Currently, there are, however, still no effective measures for the prevention and treatment of T-2 toxin. Since oxidative stress is the main mechanism of its toxicity, the use of antioxidant may be a new prevention and treatment measure.

Selenium (Se) [17, 18] is an important nutrient trace element, which plays an important role in various biological and physiological processes of animals, such as antioxidant defense, anti-cancer effect and detoxification function [19–21]. Two major Se sources, which are inorganic (selenite or selenate) and organic Se (selenomethionine) [22]. Among them, selenomethionine (SeMet) has relatively small toxicity, good safety, high bioavailability and effective biological function compared with inorganic Se, so it is considered as a dominant Se supplement and is also widely used in feed production. SeMet is an effective antioxidant, mainly through the role of selenium-containing proteins such as oxidoreductase GSH-Px and Thioredoxin Reductase (TrxR) [23]. GSH-Px and TrxR are mainly involved in the process of reducing hydrogen peroxide and lipid peroxides in the body [24]. Se, as their active center, plays a key role in the enzyme activity of GSH-Px and TrxR, thereby protecting cell membranes and organelle membranes from interference and damage by oxygen free radicals. In addition, selenium can partially antagonize the toxic effects of T-2 toxins. In chondrocytes, selenium can block T-2 toxin-induced chondrocyte apoptosis by reducing the Bcl-2-Associated X (Bax)/B-cell lymphoma-2 (Bcl-2) ratio [25]. In chicken primary liver cells, SeMet can effectively alleviate hepatotoxicity caused by T-2 toxins by increasing the level of antioxidant stress [26]. Compared with other antioxidants, SeMet as a feed additive is a natural form of existence in grain, which is safer and has a low economic cost and is suitable for large-scale use. However, the protective effect of selenium on T-2 toxin-induced liver damage in rabbits has not been reported. In this study, we established poisoning model of T-2 toxin in rabbits to investigate the effects of different doses of SeMet on T-2 toxin-induced liver injury. Furthermore, the effect of SeMet on oxidative stress and apoptosis induced by T-2 toxin was further analyzed to explore its specific protective mechanism. The results of this research may be helpful for the detoxification of T-2 toxin-contaminated feed.

## Results

### Serum biochemical analysis

**Fig. 1:**
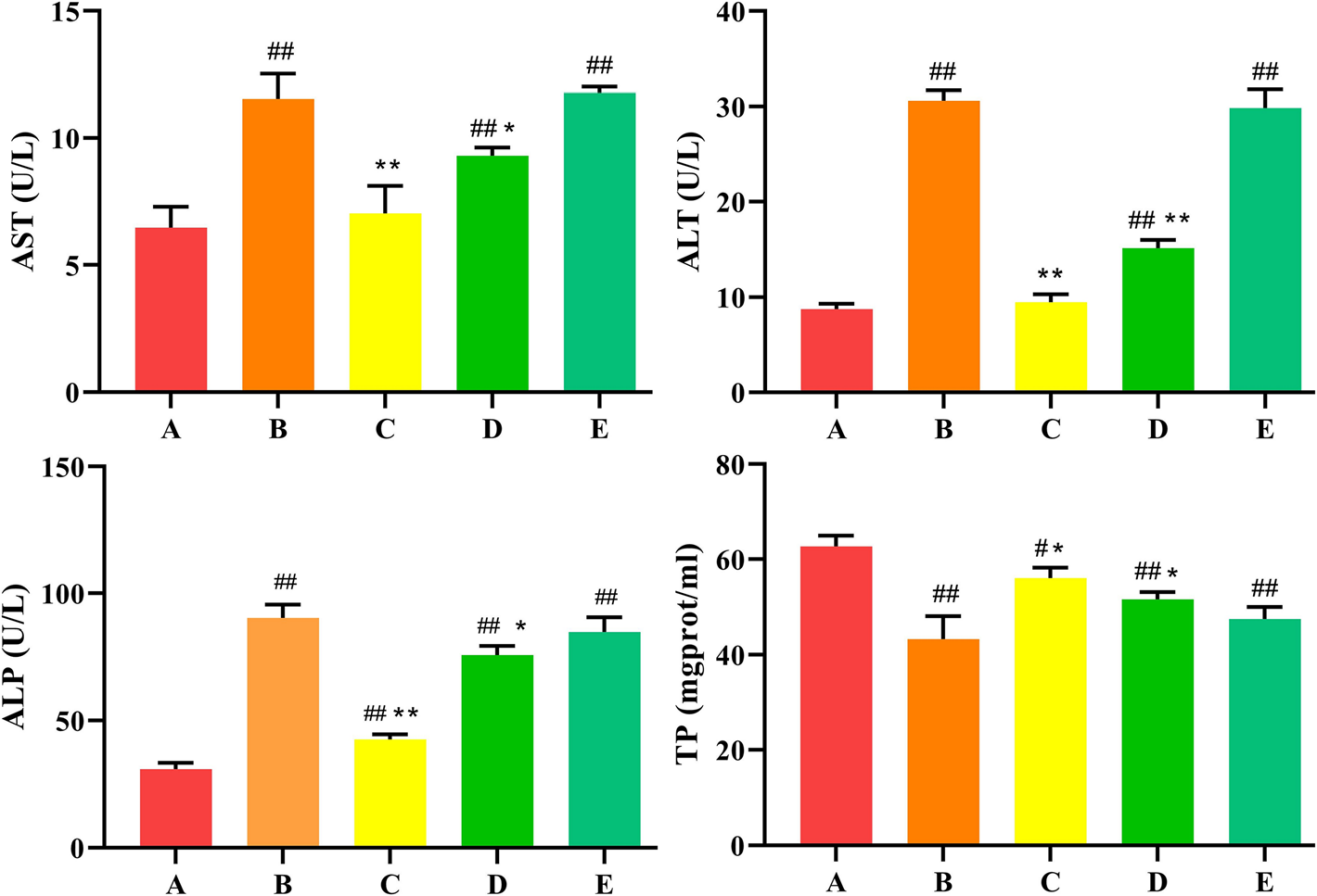
Effects of SeMet at different doses on ALT, AST, ALP, and TP levels in serum of rabbits after T-2 toxin treatment. A: control group, B: T-2 toxin group C: low-dose SeMet+T-2 toxin group, D: medium-dose SeMet+T-2 toxin group, E: high-dose SeMet+T-2 toxin group. Each value is the mean ± SE of five independent determinations. Significant differences were #*P* < 0.05 or ##*P* < 0.01 compared to control group; *P < 0.05 or **P < 0.01 compared to T-2 toxin group

To measure changes in liver function, serum levels of AST, ALT, ALP, and TP were measured in each group. After 5 days of T-2 toxin administration, the serum levels of AST, ALT and ALP in T-2 toxin-treated group were obviously higher than that in the control group, whereas the level of TP was reverse (*P* < 0.01) (Fig. 1). In the low-dose and medium-dose SeMet+T-2 toxin group, we found that activities of AST, ALT, and ALP decreased to different degrees compared with the T-2 toxin group, and TP levels also increased in varying degrees (Fig. 1). Notably, the low-dose SeMet +T-2 toxin group showed the most significant change (*P* < 0.01), and there was no significant difference between the high-dose SeMet+T-2 toxin group and the T-2 toxin-treated group (Fig. 1).

### SeMet improves T-2 toxin-induced liver pathological changes

**Fig. 2:**
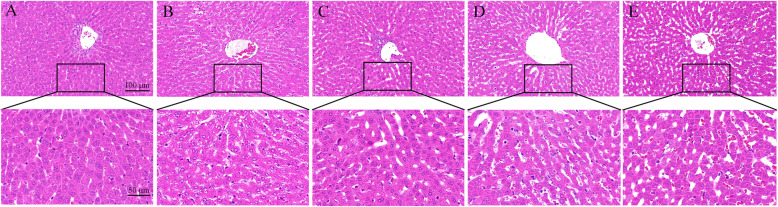
Morphological observation of liver. In the control group, hepatocytes and hepatic sinuses were neatly arranged around the central vein, and the structure was complete. Unclear outline of liver cells, disordered arrangement, severe cytoplasmic vacuolation, and fragmented nuclei were observed in the T-2 toxin treated group. After low-dose SeMet pretreatment, the liver morphology improved significantly. However, as the dose of SeMet increased, the sinusoids in the medium and high-dose SeMet + T-2 toxin groups increased significantly and accompanied by significant bleeding. **a**: control group, **b** T-2 toxin group **c**: low-dose SeMet+T-2 toxin group, **d**: medium-dose SeMet+T-2 toxin group, **e**: high-dose SeMet+T-2 toxin group. The magnification of the first row of pictures is 200× and the scale is 100 μm. The second row is a partial magnification of the first row of pictures with a magnification of 400× and a scale of 50 μm

**Fig. 3:**
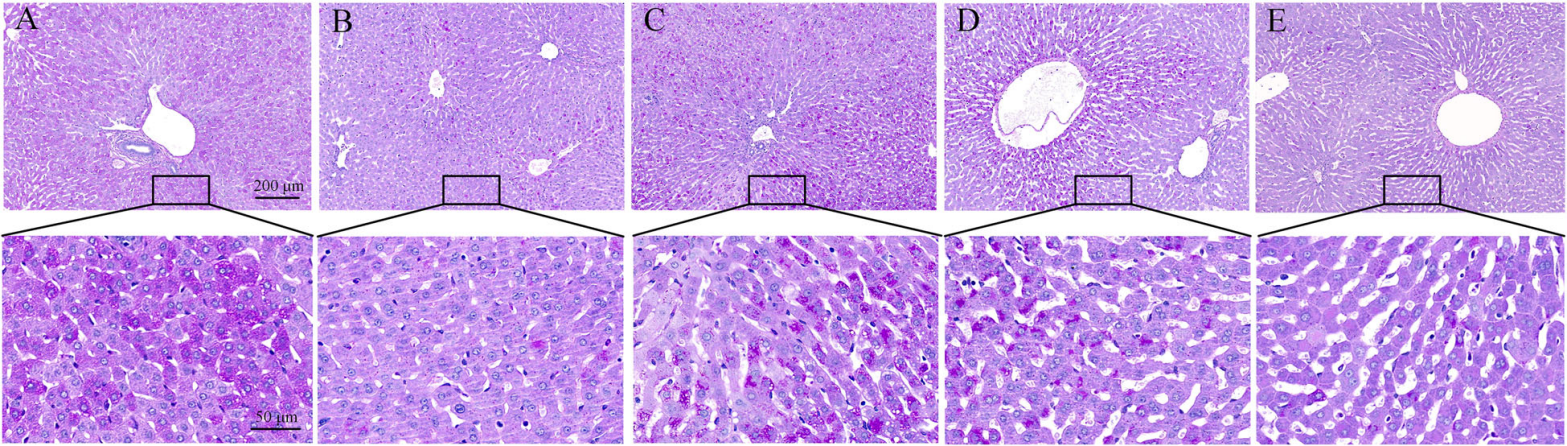
Observation of liver glycogen content changes. Hepatic glycogen was stained purple-red with PAS staining. The liver of the control group contained a large amount of liver glycogen, and the content of liver glycogen in the T-2 toxin group was significantly reduced. After low-dose SeMet pretreatment, liver glycogen increased significantly. However, hepatic glycogen decreased gradually in the middle and high dose SeMet + T-2 toxin groups. **a**: control group, **b** T-2 toxin group **c**: low-dose SeMet+T-2 toxin group, **d**: medium-dose SeMet+T-2 toxin group, **e**: high-dose SeMet+T-2 toxin group. The magnification of the first row of pictures is 100× and the scale is 200 μm. The second row is a partial magnification of the first row of pictures with a magnification of 400× and a scale of 50 μm

Histopathological analysis was performed using H&E and PAS staining of the liver sections. In the liver of the control rabbits, liver cells and hepatic sinusoids were radically arranged around the central vein with a complete structure, large and round nuclei (Fig. 2a), and a large amount of liver glycogen (Fig. 3a). Compared with the control group, unclear outlines, disordered arrangement, severe cytoplasmic vacuolation, fragmented nuclei (Fig. 2b), and significantly reduced glycogen (Fig. 3b) were found in the T-2 toxin-treated group. After low-dose SeMet pretreatment, liver tissue morphology was significantly improved (Fig. 2c), and glycogen synthesis was increased (Fig. 3c). However, with the increase of SeMet dose, the hepatic sinuses were significantly enlarged and accompanied by significant bleeding (Fig. 2d, e). Moreover, glycogen synthesis was gradually decreased with the increase of SeMet dose (Fig. 3d, e).

### SeMet suppresses T-2 toxin-induced liver oxidative stress

**Fig. 4:**
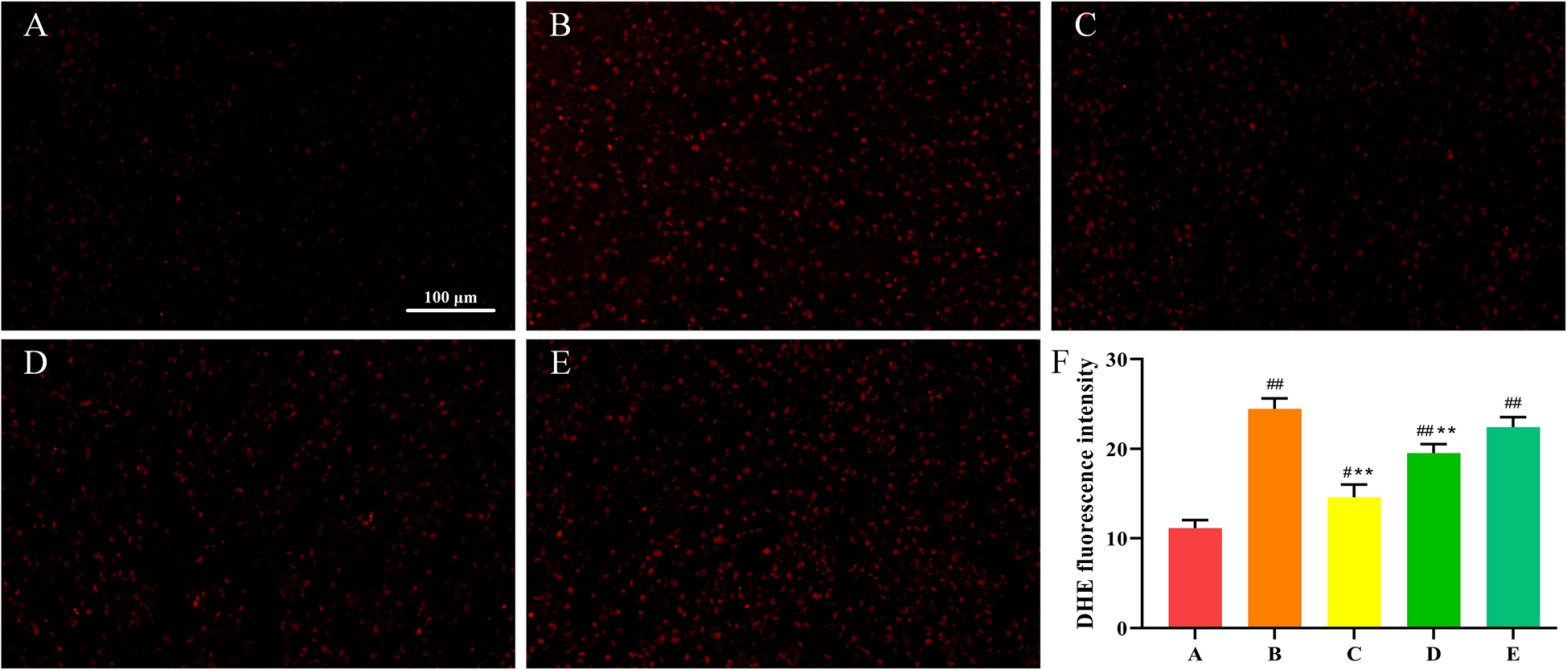
ROS expression in liver. The intensity of the red fluorescence reflects the level of ROS. Compared with the control group, the red fluorescence intensity of the T-2 toxin group was significantly enhanced, indicating that the ROS level was significantly increased. After low-dose SeMet pretreatment, ROS levels decreased significantly. However, as the SeMet dose increased, ROS levels in the middle and high-dose groups gradually increased. **a**: control group, **b** T-2 toxin group **c**: low-dose SeMet+T-2 toxin group, **d**: medium-dose SeMet+T-2 toxin group, **e**: high-dose SeMet+T-2 toxin group. **f**: Quantitative analysis of fluorescence intensity of DHE staining using imagej software. Each value is the mean ± SE of five independent determinations. Significant differences were #*P <* 0.05 or ##*P <* 0.01 compared to control group; *P < 0.05 or **P < 0.01 compared to T-2 toxin group. Magnification is 200× and scale is 100 μm

**Fig. 5:**
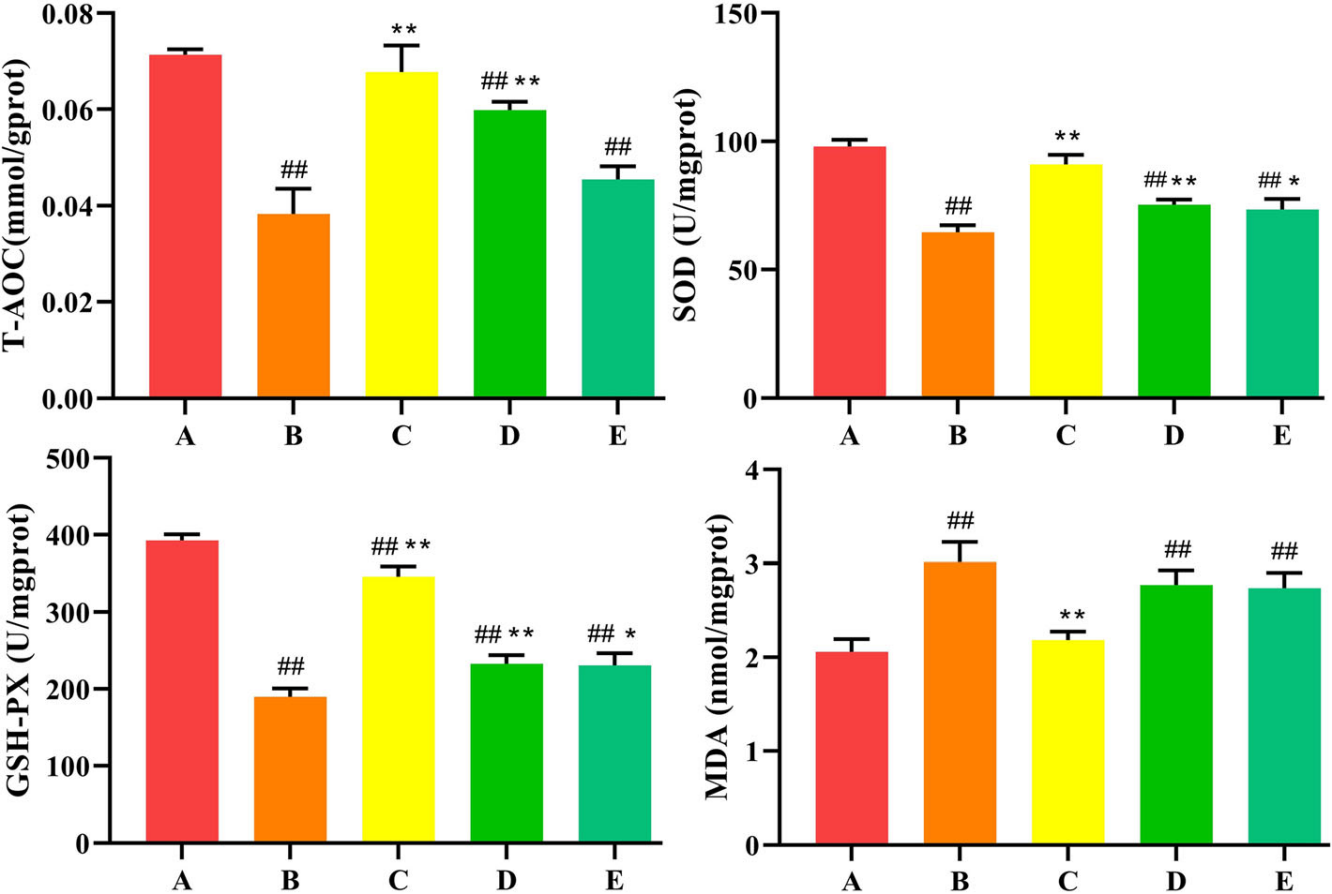
Effects of SeMet on oxidative stress markers in rabbits after T-2 toxin treatment. A: control group, B T-2 toxin group C: low-dose SeMet+T-2 toxin group, D: medium-dose SeMet+T-2 toxin group, E: high-dose SeMet+T-2 toxin group. Each value is the mean ± SE of five independent determinations. Significant differences were #*P <* 0.05 or ##*P <* 0.01 compared to control group; *P < 0.05 or **P < 0.01 compared to T-2 toxin group

In order to explore whether SeMet protects T-2 toxin-induced liver damage through antioxidant effects. DHE staining was used to detect the levels of ROS. The intensity of the red fluorescence reflected the level of ROS. As shown in Fig. 4, compared with the control group, the fluorescence intensity of the T-2 toxin group was significantly enhanced, indicating that the ROS level was significantly increased. After low-dose SeMet pretreatment, ROS levels decreased significantly, however, with the increase of SeMet dose, ROS levels increased gradually. It indicated that low dose of SeMet inhibited T-2 toxin-induced ROS production. Subsequently, the levels of T-AOC, SOD, GSH-Px, and MDA in liver tissues of each group were detected using the corresponding kits. As shown in Fig. 5, compared with the control group, the levels of T-AOC, SOD, GSH-Px in the T-2 toxin-treated group were extremely decreased (*P* < 0.01), and the levels of MDA were extremely increased (*P <* 0.01). After pretreatment with low-dose SeMet, the levels of T-AOC, SOD, GSH-Px increased significantly compared with the T-2 toxin group (*P <* 0.01), and the levels of MDA decreased significantly (*P <* 0.01). However, with the increase of SeMet dose, the levels of T-AOC, SOD, GSH-Px gradually decreased, and the level of MDA gradually increased. And there was no difference between the high-dose SeMet T-2 toxin group and the T-2 toxin group.

### SeMet inhibits T-2 toxin-induced hepatocyte apoptosis

**Fig. 6:**
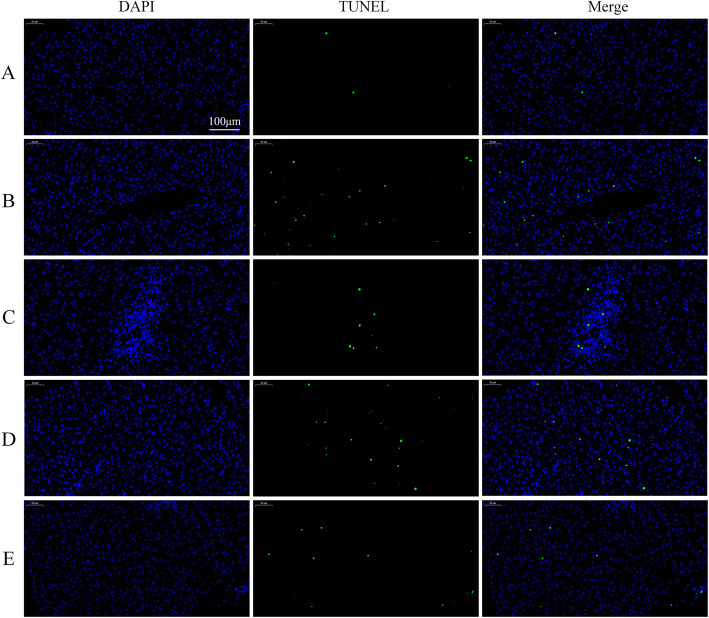
SeMet inhibits T-2 toxin-induced hepatocyte apoptosis. Nuclei of apoptotic cells were labeled with green fluorescence by TUNEL staining. All the nuclei were stained blue by DAPI and the nuclei of apoptotic cells were stained green by TUNEL. Compared with the control group, the number of apoptotic cells in the T-2 toxin group increased significantly. After low-dose SeMet pretreatment, the number of apoptotic cells decreased significantly. However, as the dose of SeMet increased, the number of apoptotic cells in the medium-dose and high-dose groups gradually increased. **a**: control group, **b** T-2 toxin group **c**: low-dose SeMet+T-2 toxin group, **d**: medium-dose SeMet+T-2 toxin group, **e**: high-dose SeMet+T-2 toxin group. Magnification is 250× and scale is 100 μm

When the cell undergoes apoptosis, the DNA strand breaks and the 3′-end is leaked out. The TUNEL staining is used to mark the broken 3′-end of the DNA to detect cell apoptosis. The number of green fluorescence represents the number of apoptotic cells. As shown in Fig. 6, the number of apoptotic cells in the T-2 toxin group was significantly increased compared to the control group. After low-dose SeMet pretreatment, the number of apoptotic cells decreased significantly. However, as the dose of SeMet increased, the number of apoptotic cells increased gradually.

### Effects of SeMet on T-2 toxin-induced hepatocyte apoptosis-related genes and proteins expression

**Fig. 7:**
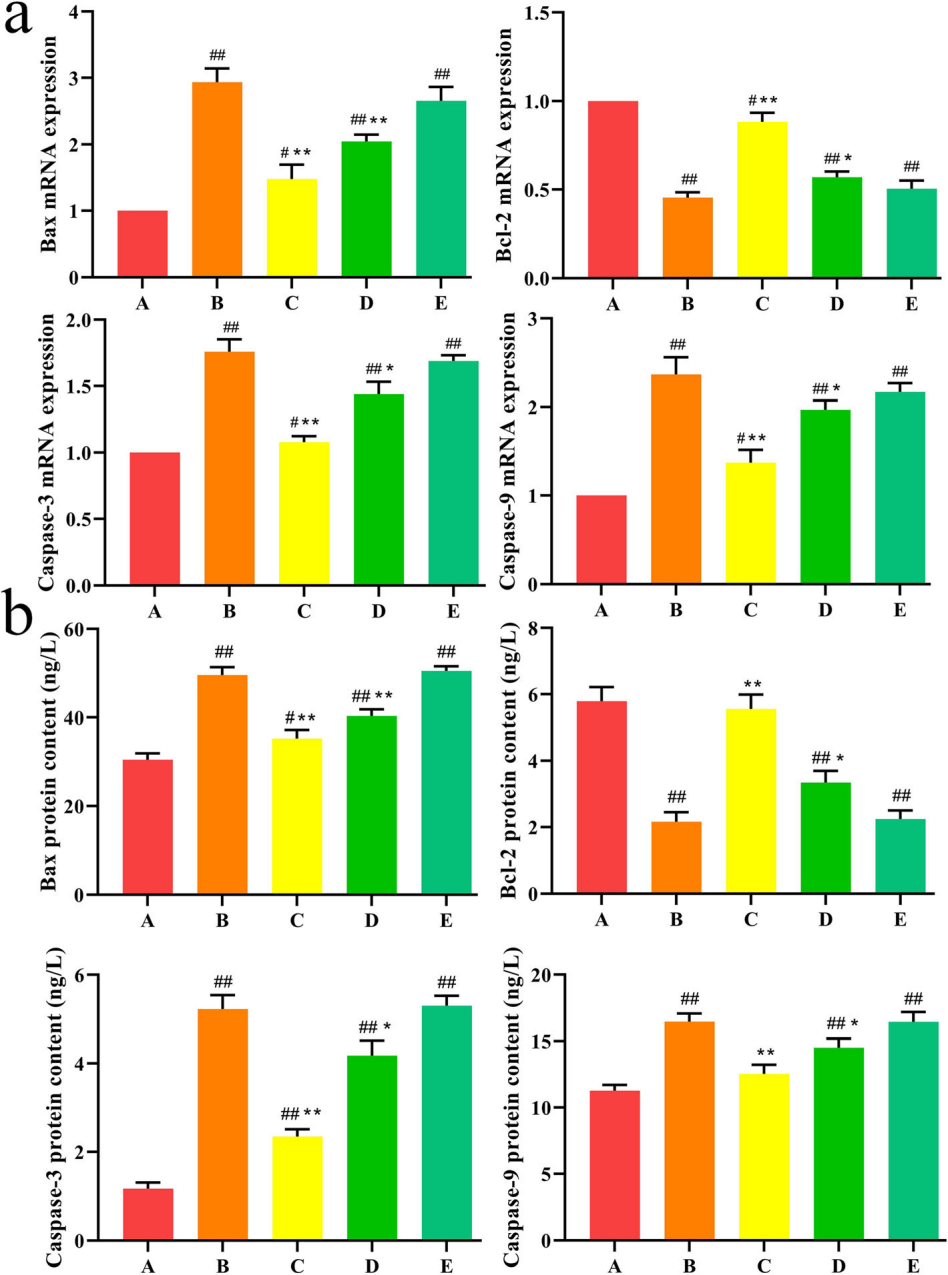
**a** Effect of SeMet on Bax, Bcl-2, Caspase-3, Caspase-9 mRNA expression in rabbits after T-2 toxin treatment. **b** Effects of SeMet on Bax, Bcl-2, Caspase-3, Caspase-9 protein expression in rabbits after T-2 toxin treatment. A: control group, B T-2 toxin group C: low-dose SeMet+T-2 toxin group, D: medium-dose SeMet+T-2 toxin group, E: high-dose SeMet+T-2 toxin group. Each value is the mean ± SE of five independent determinations. Significant differences were #*P <* 0.05 or ##*P <* 0.01 compared to control group; *P < 0.05 or **P < 0.01 compared to T-2 toxin group

In the execution and regulation of apoptosis, the Bcl-2 family and the Caspase family play an indispensable role. We used qPCR and ELISA to detect the gene and protein expression levels of pro-apoptotic gene Bax, anti-apoptotic gene Bcl-2, and proteases Caspase-3 and Caspase-9 in each group. As shown in Fig. 7, compared with the control group, the expression levels of Bax, Caspase-3, and Caspase-9 in the T-2 toxin-treated group were significantly increased (*P* < 0.01), and the expression levels of Bcl-2 were significantly reduced (*P <* 0.01). After low-dose SeMet pretreatment, the expression levels of Bax, Caspase-3, and Caspase-9 were significantly reduced (*P <* 0.01), and the expression levels of Bcl-2 were significantly increased (*P <* 0.01). However, as the SeMet dose increased, the expression levels of Bax, Caspase-3, and Caspase-9 gradually increased, and the expression levels of Bcl-2 gradually decreased. And there was no difference between the T-2 toxin group and the high-dose SeMet group. These results indicate that SeMet at the low and medium doses can reduce the increase of Bax, Caspase-3, and Caspase-9 expression in liver cells induced by T-2 toxin, and increase Bcl-2 expression levels. Moreover, the effect of SeMet at lower doses is more obvious.

## Discussion

SeMet has been widely used as a feed additive to prevent animal poisoning due to its excellent antioxidant capacity, high bioavailability and low toxicity [27, 28]. In this study, in order to investigate the effects of SeMet on T-2 toxin-induced liver injury in rabbits, we added low, medium, and high doses of SeMet to the diet for 21 days, and on the 17th day oral T-2 toxin for five consecutive days. The results showed that after oral administration of T-2 toxin, the enzyme activities of AST, ALT and ALP in rabbit serum increased significantly, indicating that liver cells were damaged, and HE staining also showed that hepatocytes were disordered and severe vacuole degeneration occurred. Moreover, the TP content and liver glycogen in the T-2 toxin-treated group were significantly reduced, suggesting that liver function was impaired. After low-dose SeMet pretreatment, liver damage was significantly repaired, and the ability of liver glycogen and protein synthesis was significantly improved. However, the repair effect of SeMet was not dose-dependent. The results showed that the low-dose SeMet had the best repair effect. With the increase of the dose, the protective effect of SeMet was significantly weakened, and the high-dose SeMet had no this role. The reason for this result may be due to the amount of selenium added. Selenium is an indispensable trace element in animals. Animals ingesting different doses of selenium will play different roles in growth and development [17, 18]. The lack of selenium in animals will reduce the activity of GSH-Px in blood and tissues, which will reduce the animal’s immune function and defensive capability. Moderate selenium supplementation can scavenge oxygen free radicals and play an antioxidant role, promote animal growth, and improve animal performance. However, when animals consume excessive selenium, selenium will produce free radicals and cause toxic effects on the body. For example, after feeding rats with sodium selenite for 6 weeks, it was found that high concentration of sodium selenite would cause liver fatty acid β oxidation, provide electrons for mitochondrial oxidative phosphorylation, and lead to excessive production of ROS, thereby causing disturbance of glucose and lipid metabolism, leading to the occurrence of liver insulin resistance [29]. Li Jian et al. [30] also found that high concentration of sodium selenite can increase the ROS level in human acute promyelocytic leukemia cell line NB4 cells, causing oxidative stress and leading to cell apoptosis. In this study, the medium dose of SeMet may have exceeded the minimum dose that can cause selenium poisoning in rabbits. However, there has been no report on the dosage of selenium poisoning in rabbits, which will be the focus of our future research.

Oxidative stress was defined as the lack of balance between the occurrence of reactive oxygen species (ROS) and the organism’s capacity to counteract their action by the antioxidative protection systems [31, 32]. The occurrence of oxidative stress may be due to excessive production of ROS, or a decrease in cellular antioxidant levels [33]. Under normal physiological conditions, the body can rely on its own antioxidant enzymes, such as: SOD, GSH-Px, CAT to maintain the redox balance in the cells to protect the body tissues and cells to prevent ROS damage [34]. When the body is subjected to various harmful stimuli, the cells produce excessive ROS and cause oxidative damage to the cells, such as DNA damage, protein oxidation, and lipid peroxidation [35, 36]. Recent studies have shown that oxidative stress is an important mechanism for T-2 toxins to exert cytotoxicity and can cause damage to many cells, including rat ovarian granulose cells [37], THP-1 monocytes [38], and chicken growth plate chondrocytes [39]. Our study also showed that T-2 toxin significantly increased the content of ROS and MDA, and significantly reduced the activity of T-AOC, antioxidant enzymes SOD and GSH-Px, causing oxidative stress in liver cells. SeMet is an excellent antioxidant. Our study found that low-dose SeMet can significantly reduce the oxidative stress level after T-2 treatment in rabbits. However, with the increase of SeMet dose, the ROS level was increased instead of further decreased, and the activity of antioxidant enzymes SOD and GSH-Px was decreased, which further increased the oxidative stress level. It is suggested that low-dose SeMet may play a protective role through its excellent antioxidant capacity. However, higher doses of SeMet cause a toxic effect through oxidative stress.

**Fig. 8:**
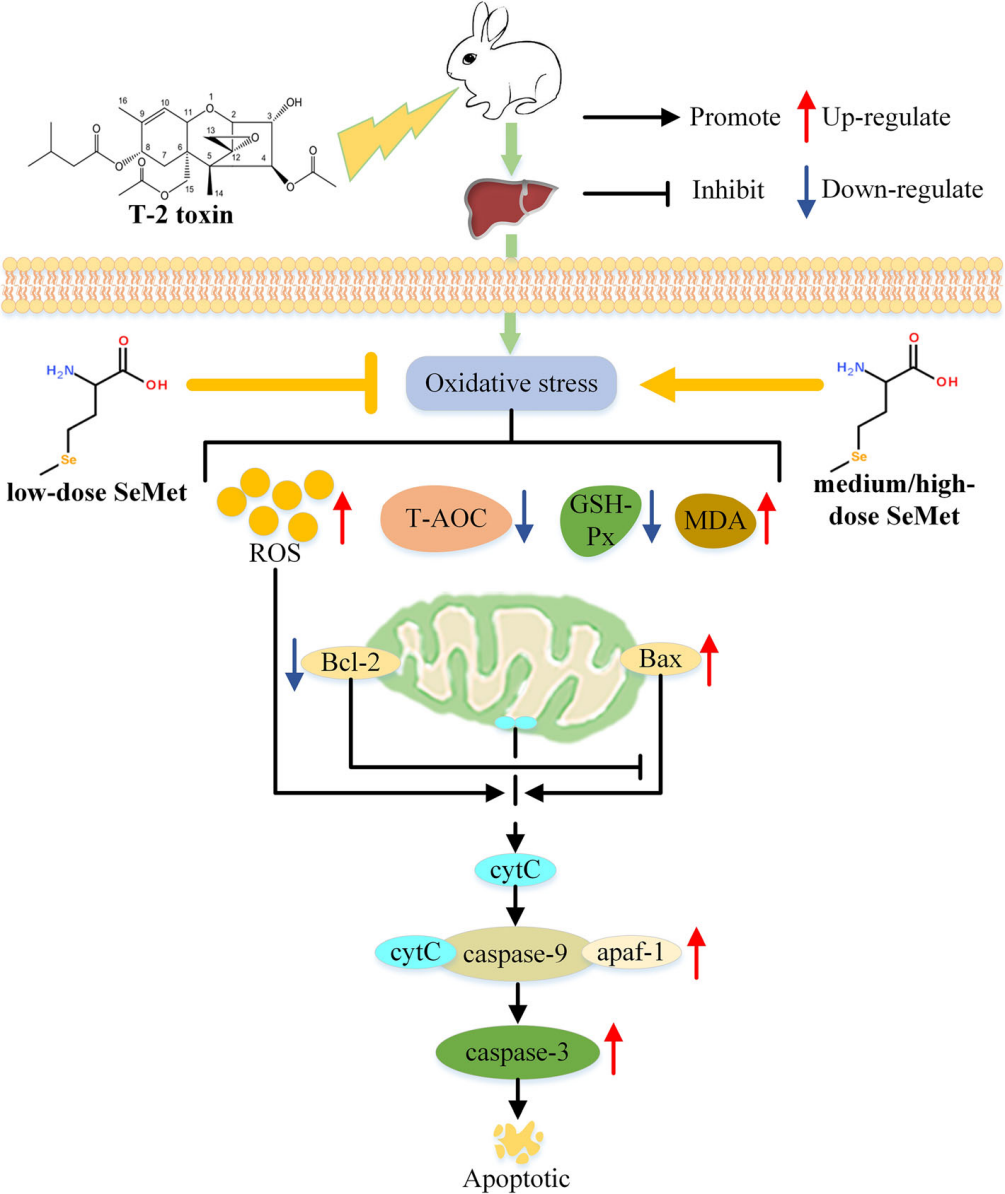
A schematic diagram of the proposed mechanisms by which SeMet ameliorates T-2 toxin-induced liver injury by inhibiting ROS-induced mitochondrial-caspase apoptosis pathway. R0S: reactive oxygen species, T-AOC: total antioxidant capacity, GSH-Px: glutathione peroxidase, MDA: malondialdehyde, SeMet: selenomethionine, Bcl-2: B-cell lymphoma-2, Bax: Bcl-2-Associated X, cytC: Cytochrome C, apaf-1: apoptotic protease activating factor 1

It is well known that mitochondria are the most vulnerable targets of ROS, and oxidative stress can cause cell death by triggering the mitochondrial-caspase apoptosis pathway [40]. After oxidative stress occurs in cells, excess ROS can directly or indirectly damage the mitochondrial membrane, reduce the mitochondrial membrane potential, and promote the release of the apoptosis factor Cytochrome C (cytC) from the mitochondrial membrane space into the cytoplasm (Fig. 8). The release of cytC is regulated by members of the Bcl-2 family such as Bcl-2 and Bax. Bax is a protein located in mitochondria. After activation, it can form pore complex in the outer membrane of mitochondria through polycondensation to promote the release of cytC. However, Bcl-2 can inhibit the pro-apoptotic effect of Bax, so the ratio of Bax/Bcl-2 plays an important role in determining whether cells go toward apoptosis [41]. After entering the cytoplasm, cytC will form apoptotic body with apoptotic protease activating factor 1 (apaf-1) and caspase-9, further activating caspase-3. Eventually the apoptotic process is activated (Fig. 8). In this study, the results of TUNEL staining showed that T-2 toxin induced hepatocyte apoptosis, and low-dose SeMet significantly reduced the number of apoptotic cells. To further investigate the protective mechanism of low-dose SeMet, we further examined the expression of several key genes in the mitochondrial-caspase apoptotic pathway. The results showed that the mRNA and protein expression of Bax, caspase-3 and caspase-9 were significantly decreased and the expression of Bcl-2 was increased. We speculate that low-dose SeMet is likely to protect liver cells from the toxicity of T-2 toxin by inhibiting the mitochondrial-caspase apoptosis pathway. However, further experiments are needed to confirm, which will be the focus of our next research.

In conclusion, the results showed that low-dose SeMet could effectively protect rabbits from T-2 toxin-induced liver injury, and its protective mechanism may involve the inhibition of ROS-induced mitochondrial-caspase apoptosis pathway. Our study provides a theoretical basis for SeMet to prevent T-2 toxin poisoning in rabbits. In fact, T-2 toxin pollution not only brings huge economic losses to the rabbit breeding industry, but also seriously affects the poultry breeding. Poultry are sensitive to T-2 toxin, which can cause a variety of toxic effects when ingested by poultry, and there is currently no effective preventive measures [42]. According to the results of this study, we speculate that SeMet may also play a role in the prevention of T-2 toxin poisoning in poultry. Further experiments are needed for research.

## Conclusions

The current study provides information on the protective effect of SeMet on T-2-toxin-induced liver injury in rabbit and explore its molecular mechanism. The results showed that low-dose SeMet significantly improved T-2 toxin-induced liver injury. To further investigate the protective mechanism of low-dose SeMet, we found that low-dose SeMet can reduce the level of oxidative stress and the number of hepatocyte apoptosis. Moreover, the levels of Bax, caspase-3 and caspase-9 were significantly reduced and the levels of Bcl-2 were increased. Therefore, we confirmed that low-dose SeMet may protect rabbit hepatocytes from T-2 toxin by inhibiting the mitochondrial-caspase apoptosis pathway.

## Materials and methods

### Chemicals and reagents

T-2 toxin and SeMet was purchased from Sangon Biotich (Shanghai, China). ALT, AST, TP, Alkaline phosphatase (ALP), SOD, GSH-PX, Malondialdehyde (MDA), T-AOC assay kits and bicinchoninic acid (BCA) protein assay kits were purchased from the Jiancheng Bioengineering Institute (Nanjing, China). PrimeScript™ RT reagent kit (Perfect Real Time) and SYBR® Premix Ex Taq kits were purchased from Takara (Dalian, China). All other chemicals used were of the highest grade commercially available.

### Animals and treatments

Fifty male New Zealand rabbits (30 d, 0.5 ± 0.1 kg) from the rabbit farm of Muzhichun Animal Husbandry, JiYuan, China were placed in a breeding room at 21–25 °C and a relative humidity of 50 to 70%. Allow them to eat and drink freely. After adaptive feeding for 1 week, the rabbits (30 d, 0.4 ~ 0.6 kg) were randomly divided into 5 groups, 10 in each group, by using of Excel software based on the body weight, namely the control group (A), the T-2 toxin group (B) and the low, medium and high dose SeMet treatment group (C, D and E). The basic diet was based on the nutritional requirements of NRC (1977) meat rabbits, combined with the growth and development characteristics of New Zealand meat rabbits and the situation of feed resources in this area. Ingredients and chemical analyses of the basal diet are presented in Table 1. Group A and group B were fed with basic diet and drinking water for 21 days, while groups C, D and E were fed feeds containing 0.2 mg/kg, 0.4 mg/kg and 0.6 mg/kg selenium (Dissolve the SeMet in water, spray it evenly on the feed with a spray pot, then dry it for later use) for 21 days, respectively. On day 17, each rabbit in group A began to take 1 ml olive oil orally every day for 5 days, and each rabbit in group B, C, D and E began to take 0.4 mg/kg of T-2 toxin orally every day for 5 days [8]. (T-2 toxin was dissolved in 1 ml of olive oil). Rabbits were euthanized 24 h following the last administration. The liver was removed immediately after the rabbit was executed. All procedures used in this study were approved by the Ethics Committee of Henan University of Science and Technology (No.20190619024).

**Table 1:** Ingredients and chemical composition of the basal diets

Feed ingredients	Percent %
Grass powder	50
Wheat bran	20
Corn	15
Soya bean meal	11
Sodium chloride	1.5
Dicalcium phosphate	1.0
Limestone	1.0
Vitamin–mineral premixa	0.5
Total 100	100
Calculated composition	
Digestable energy	26.50 MJ/kg
Crude Protein (%)	16.5
Crude Fiber (%)	5.0
Calcium (%)	0.9
Phosphorus (%)	0.63

### Biochemical assays

After the rabbit was sacrificed, blood was immediately drawn from the heart. Blood samples were centrifuged at 3500 rpm for 10 min at 4 °C, and serums were isolated immediately after collection. The serum samples were stored at − 20 °C and the activities of AST, ALT, TP, and ALP in the serum were measured using a commercial kit according to the operating instructions [43].

### Histopathological analysis

Liver tissue samples were fixed in 4% paraformaldehyde. After fixation for 48 h, tissues were dehydrated, paraffin embedded, sectioned at 5 μm, and stained with haematoxylin and eosin (H.E) for histological examination [43]. Three representative sections from each liver were detected. Ten high-power fields (× 200) per section were examined for each sample.

### Periodic acid-Schiff (PAS) staining

The prepared paraffin sections were stained with PAS. The sections were dewaxed with xylene and rehydrated ethanol gradient. After treatment with 1% periodic acid (Servicebio) for 15 min, the sections were washed under running tap water, washed twice with distilled water and immersed in Schiff’s reagent (Servicebio) for 30 min under protection against exposure to light. The tissues were stained with haematoxylin, differentiated, dehydrated and mounted. Images were obtained under a computer-supported imaging system connected to a light microscope (Olympus AX70) [43, 44].

### Measurement of ROS

DHE stainings were performed essentially as previously described [31]. Frozen liver sections were stained with DHE (10 mM) at 37 °C for 30 min [45]. Fluorescence was visualized by con-focal microscopy (LSM 710, Zeiss). Quantitative analysis of fluorescence intensity of DHE staining using imagej software (version 1.47; National Institutes of Health, USA) [43, 44, 46].

### Measurement of oxidative stress markers

MDA, SOD, T-AOC, GSH-Px levels were detected using specific assay kits according to the manufacturer’s instructions. Each test was measured with 5 duplicate samples [44].

### Terminal dUTP Nick-end labeling (TUNEL) assay

Following the protocol of manufacturer, TUNEL staining was conducted by an in situ cell death detection kit (DeadEnd Fluorometric TUNEL System, Promega, Madison, USA). The nuclei of all cells were counter-stained with DAPI. The number of TUNEL and DAPI positive nuclei was counted in six images that were chosen randomly from non-overlapping areas of each group. The data were presented as the percentage of TUNEL positive cells [47].

### Enzyme-linked immunosorbent assay

The protein content of apoptosis-related factors Bax, Bcl-2, Caspace-3, and Caspace-9 in rabbit liver was measured using enzyme-linked immunosorbent assay (ELISA) kits. Liver tissues were prepared following the manufacturer’s instructions. Briefly, tissue samples were split, centrifuged, and added into the specimen diluent. After 60 min of incubation, the contents of the wells were decanted, washed and substrate was added which develops a blue color in the presence of enzyme. The optical density (OD) was assessed at 450 nm by microplate reader (Bio-Rad, USA). The concentrations of Bcl-2, Bax, caspase-3 and caspase-9 were calculated using Curve Expert 1.3 [43, 44].

### Quantitative real-time reverse transcription PCR (RT-qPCR)

The liver tissues were subjected to qRT-PCR analysis. RNA extraction and real-time PCR were performed as described in previous studies in our laboratory. Briefly, the total RNA from frozen liver tissues was extracted using TRIzol reagent (Cwbio Technologies, Beijing, China). cDNA was generated using the PrimeScript RT reagent kit with cDNA eraser (Takara, Dalian, China). Quantitative PCR assays were carried out using the SYBR® Premix Ex Taq™ kit (Takara, Dalian, China). Target gene expression was quantified using the 2^**-△△**^Ct method and normalized to the expression of GAPDH. The primer sequences are summarized in Table 2 [43–45].

**Table 2:** Primer sequences used in RT-PCR

Gene	Primer sequence (5′–3′)
Bcl-2	Forward: GACGACTTCTCCCGGCGCTA
	Reverse: ACACATGACCCCACCGAAC
Bax	Forward: CACCAAGAAGCTGAGCGAGT
	Reverse: GCAAAGTAAAACAGGGCGACA
Caspase-3	Forward: AGATGTAAATGCAGCAAACCTC
	Reverse: TCCTTCATCACCGTGGCTT
Caspase-9	Forward: ACATCCTCGTGTCCTACTCC
	Reverse: TTGTAAATCCCTCGCTCGGAA
GAPDH	Forward: GTTGTCGCCATCAATGATCCA
	Reverse: TTCCCGTTCTCAGCCTTGACC

### Statistical analysis

The data were analyzed with one-way ANOVA and LSD’s (Least Significant Difference) post hoctest via SPSS 20.0 statistical software. All the data were presented as means ± SD. Statistical significance was considered when *p* < 0.05.

## Data Availability

The data underlying this article will be shared on reasonable request to the corresponding author.

## Abbreviations

R0S: Reactive oxygen species
T-AOC: Total antioxidant capacity
GSH-Px: Glutathione peroxidase
MDA: Malondialdehyde
SeMet: Selenomethionine
Bcl-2: B-cell lymphoma-2
Bax: Bcl-2-Associated X
cytC: Cytochrome C
apaf-1: Apoptotic protease activating factor 1
